# *GW*‐BSE Calculations of Electronic Band Gap and Optical Spectrum of ZnFe_2_O_4_: Effect of Cation Distribution and Spin Configuration

**DOI:** 10.1002/cphc.201901088

**Published:** 2020-02-12

**Authors:** Anna C. Ulpe, Thomas Bredow

**Affiliations:** ^1^ Mulliken Center for Theoretical Chemistry, Institut für Physikalische und Theoretische Chemie Universität Bonn Beringstraße 4–6 D-53115 Bonn Germany

**Keywords:** Perturbation Theory, Spinel Ferrites, Ab Initio Calculations, GW calculations, Photochemistry

## Abstract

The *G*0*W*0, ev*GW*0, ev*GW*, and sc*GW*0 approximations to many‐body perturbation theory combined with the Bethe‐Salpeter approach (BSE) are applied to calculate electronic and optical properties of the open‐shell spinel ferrite ZnFe_2_O_4_. The effect of the various degrees of self‐consistency is assessed by comparison to recent experimental results. Furthermore, the influence of the method for obtaining the ground‐state wavefunction is studied, including the GGA functional PBE with and without an intermediate step using the COHSEX approximation, as well as PBE+*U*, where we try to minimize the influence of the Hubbard potential *U*. Best agreement for the optical band gap and the first maxima of the excitation spectrum is obtained with the ev*GW* method based on a PBE+*U* wavefunction. This method is chosen and converged carefully to yield quantitative results for the optical spectra of four different magnetic structures and cation distributions of ZnFe_2_O_4_. With the results we provide a possible explanation for inconsistency in experimental results.

## Graphical abstract

**A theoretical approach** is developed to obtain qualitative information on the optical properties of the spinel zinc ferrite ZnFe_2_O_4_. The calculations reveal a pronounced dependency of the optical spectrum of ZnFe_2_O_4_ on the cation and magnetic state.

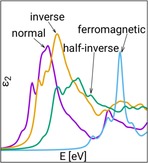

## 1 Introduction

As a consequence of the climate change, the urge for sustainable energy production from renewable sources has increased dramatically during the last years. A possible solution to this problem is photoelectrochemical water splitting, where the main issue is the search for suitable electrode materials. Spinel ferrites *M*Fe_2_O_4_, where *M* is a divalent cation, are possible candidates for this purpose.^1^, ^2^ In the spinel structure, spacegroup Fd$\bar{3}$ m, the oxygen atoms form a cubic close‐packed lattice and the cations *M* and Fe occupy 1/8 of the tetrahedral and 1/2 of the octahedral positions. When the tetrahedral sites are occupied by *M* only, the spinel is denoted as ‘normal’. In a fully inverse spinel, tetrahedral sites are occupied by Fe only, while *M* and the remaining Fe atoms are distributed in octahedral sites.

Experimentally measured optical band gaps of ZnFe_2_O_4_ show a large variation depending on the spectroscopic method and on details of the preparation.^3^ Quantum‐chemical calculations are an alternative tool for the investigation of *M*Fe_2_O_4_ for its applicability as photocatalyst. In the calculations, defect structures, inversion, or different spin states can be studied explicitly, which in this extent is not possible for experimental studies.

However, the calculation of properties of open‐shell systems, especially transition metal oxides with strongly correlated *d*‐electrons, is problematic for density‐functional theory (DFT). Relevant properties, in particular the band gap, are often inaccurately described by standard Kohn‐Sham (KS)‐DFT calculations.^4^ Because of this, the utilization of more advanced procedures as the many‐body perturbation theory within the *GW* formalism^5^ is mandatory.

However, while *GW* methods give an overall improvement of the electronic properties, e. g. the fundamental band gap, by using a quasiparticle picture, the problem of KS‐DFT to describe open‐shell systems with multi‐reference character persists. In the present study we focus on zinc ferrite (ZnFe_2_O_4_). The multi‐reference character of this compound is limited, since Zn^2+^ has a *d* ^10^ configuration and Fe^3+^ has a stable *d* ^5^ high‐spin configuration.

We compare the results of *GW* routines with varying degree of self‐consistency. The computationally least expensive G0W0 approach calculates the Green's function *G*0 from the DFT wavefunction and follows a non self‐consistent, perturbative scheme for the calculation of screened exchange in W0 . For eigenvalue (ev) *GW* and *GW*0 the quasiparticle eigenvalues are updated iteratively in the calculation of *G* (ev*GW*0) or *G* and *W* (ev*GW*). Using self‐consistent (sc) *GW* or *GW*0, a full update of the orbitals and eigenvalues is performed in *G* (sc*GW*0) or in *G* and *W* (sc*GW*).^6^ A Vertex correction is necessary to compensate the neglect of higher‐order terms in the *GW* approximation and has been shown to improve the results.^7^ Furthermore there are various simplifications of the *GW* algorithm, e. g. the static Coulomb‐Hole Screened Exchange (COHSEX) approximation.^5^

We tested the *GW* variants G0W0 , evGW0 , ev*GW* and scGW0 on zinc ferrite (ZnFe_2_O_4_) including three different initial wavefunctions, that are obtained from

- The GGA functional PBE
- The GGA functional PBE followed by a converged COHSEX calculation
- A PBE+*U* calculation with the smallest possible *U* that opens a band gap in the ground state calculation

By comparison to experiment, we aimed to identify a suitable procedure to quantitatively describe electronic and optical properties of zinc ferrite. It was shown before, that experimental results on the optical band gap of ZnFe_2_O_4_ are inconsistent.^3^ We will try to explain these inconsistencies by providing results on different cation distributions and magnetic states for ZnFe_2_O_4_.

## Computational Details

The calculations were carried out using the *Vienna Ab‐Initio Simulation Package* (VASP)^8^ version 5.4.4. PAW pseudopotentials (Zn_sv_GW, Fe_sv_GW, O_GW_new)^9^ optimized for *GW* calculations were used to describe the core electrons. The plane wave cutoff energy, number of bands, Monkhorst‐Pack grid, *GW*‐cutoff energy and the number of frequency points (NOMEGA) have been converged carefully to 600 eV, 960, 4×4×4, 200 eV and 128, respectively. Only for calculations applying the scGW0 algorithm, the cutoff energy was reduced to 450 eV in order to decrease the computation time. The size of the Monkhorst‐Pack grid and plane waves cutoff energy only has a minor effect on the calculated band gap, whereas the *GW*‐cutoff energy, the number of bands and frequency points are critical convergence parameters. Details on the convergence can be found in Tables S1 and S2 as well as Figures S1 and S2 in the supporting information (SI).

The ground state wavefunction was obtained by self‐consistent PBE or PBE+*U* calculations with blocked Davidson iteration scheme and small values of the smearing parameter (σ=0.005 ). For the Hubbard correction the simplified rotationally invariant approach introduced by Dudarev et al.^10^ was used. The PBE+*U* starting point assures that the ground state wavefunction does not have metallic character, which is the case for PBE. Since we found that the Hubbard *U* influences the *GW* calculation and hence the optical spectra, the smallest possible *U* was chosen with which a band gap in the ground state calculation opens. Another approach to obtain a non‐metallic ground state is an intermediate step using 8 cycles of COHSEX.

After that, different versions of *GW* were applied, namely G0W0 , ev*GW*0, ev*GW*, and sc*GW*0. A vertex correction was applied to the converged *GW* quasi‐particle energies. Finally, to obtain the optical gap, a calculation with the Bethe‐Salpeter formalism has been carried out. The BSE were solved for the 16 highest occupied and 16 lowest unoccupied bands.

The unit cell parameter and the atomic coordinates were optimized beforehand to account for effects of cation distribution and spin state. The structural optimization was done with the CRYSTAL17 program code,^11^ ECP basis sets^12^, ^13^, ^14^ and the PW1PW hybrid functional.^15^ It was shown previously^3^ that this approach leads to an accurate description of the zinc ferrite structure. More details on the optimization procedure can be found in Ref. [3].^]^

## 2 Results and Discussion

In the literature there is broad agreement that ZnFe_2_O_4_ is an antiferromagnetic normal spinel with a degree of inversion close to 0, hereafter referred to as configuration N‐afm.^16^ Nevertheless, other cation distributions and spin configurations are energetically close.^3^ The most important ones are ZnFe_2_O_4_ with normal cation distribution and ferromagnetic spin state, in this work denoted as N‐fm, and an inverse cation distribution with iron in tetrahedral (8a) sites coupling antiferromagnetically with iron in octahedral (16d) sites.^3^ The latter is strictly speaking a ferrimagnetic state, despite of the total magnetic moment being zero, because tetrahedral and octahedral positions are not symmetry‐equivalent. Therefore hereafter this state is referred to as I‐fim. Additionally, a half‐inverse cation distribution where ions in tetrahedral positions couple ferrimagnetically with ions in octahedral positions, was considered and is denoted as hI‐fim.

The results for the lattice parameter *a* and the fractional coordinate of oxygen *u* after geometry optimization are listed in Table 1. The relaxed *a* and *u* of all of the configurations are close to experiment. Since the experimental measurement was done at room temperature, while the calculation is carried out at 0 K, an underestimation of the lattice constant was to be expected, and is the highest with only −0.7 % for configuration I‐fim. For *u* best agreement with experiment is found for the ferromagnetic configuration N‐fm. The qualitative result is that the optimized structures are of good quality and can be used for the further calculations.

**Table 1:** Results for lattice parameter *a* and fractional coordinate of oxygen *u* after structural relaxation of different configurations of ZnFe_2_O_4_. CRYSTAL‐PW1PW results.

Configuration	*a*	*u*
N‐afm	8.418	0.2655
N‐fm	8.425	0.2602
I‐fim	8.380	0.2573
hI‐fim	8.401	0.2655
Exp^a^	8.443	0.2615

^a^Ref. [17]

**Figure 1:**
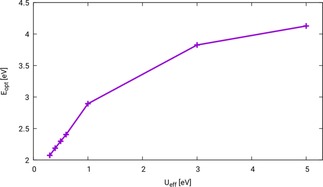
Dependence of the optical band gap $E_{\mathrm{opt}}$ of ZnFe_2_O_4_ on the Hubbard parameter *U*_eff_. Calculations are carried out with PBE+*U* (*U*_eff_=0.3, 0.4, 0.5, 0.6, 1.0, 3.0, 5.0 eV)/evGW/BSE, all calculations including vertex corrections.

The PBE+*U* wavefunction correctly corresponds to a semiconducting ground‐state provided that sufficiently large values of the Hubbard correction parameter *U* are selected. Unfortunately, the Hubbard potential influences the calculated fundamental and optical gaps, which introduces an empirical factor. Figure 1 shows the optical band gap of ZnFe_2_O_4_ configuration N‐afm calculated with PBE+*U* (*U*_eff_=0.3, 0.4, 0.5, 0.6, 1.0, 3.0, 5.0 eV)/evGW/BSE in dependence of the utilized *U*_eff_.

As can be seen, the influence of *U*_eff_ on the optical band gap is linear for $U_{\mathrm{eff}}\leq 1.0$ eV (2.07 eV for *U*_eff_=0.3, 2.18 eV for *U*_eff_=0.4, 2.29 eV for *U*_eff_=0.5, 2.40 eV for *U*_eff_=0.6 and 2.89 eV for *U*_eff_=1.0). For larger values of $U_{\mathrm{eff}}$ the increase of *E*_opt_ with increase of *U*_eff_ becomes smaller (3.83 eV for *U*_eff_=3.0 and 4.13 eV for *U*_eff_=5.0). In literature, often values between 4.0 and 5.0 eV are used for the Hubbard potential.^18^, ^19^, ^20^, ^21^ For these values the band gap of ZnFe_2_O_4_ is overestimated significantly by our approach (cf. Table 2). The PBE+*U*/evGW/BSE optical spectra that correspond to the calculations in Figure 1 can be found in the supporting information, Figure S3. It can be seen that the *U*_eff_ parameter that was chosen for the calculation of the ground‐state not only shifts the first excitation, also the shape of the spectrum changes remarkably. We conclude that with the utilization of a standard Hubbard *U* parameter the *GW*/BSE result suffers from a systematic error.

**Table 2:** Vertex‐corrected electronic band gap ($E_{g}^{elec}$ ), energy of first excited state with non‐zero oscillator strength ($E_{osz}^{non-zero}$ ), and energy of first maximum peak ($E_{osz}^{1^{st}-max}$ ) of ZnFe_2_O_4_ obtained with the different *GW*‐BSE approaches. All energies in eV.

Method	$E_{g}^{elec}$	$E_{osz}^{non-zero}$	$E_{osz}^{1^{st}-max}$
PBE/COHSEX/G0W0 /BSE	2.57	2.26	2.37
PBE/COHSEX/evGW0 /BSE	2.76	2.42	2.51
PBE/COHSEX/ev*GW*/BSE	2.84	2.49	2.57
PBE+*U* ^a^/G0W0 /BSE	2.13	1.89	1.89
PBE+*U* ^a^/evGW0 /BSE	2.38	2.02	2.02
PBE+*U* ^a^/ev*GW*/BSE	2.48	2.07	2.07
PBE/sc*GW*0/BSE	2.74	2.46	2.69
Experimental (optical) band gap	1.78−2.01^b^,2.61−3.25^c^		

^a^ *U*_eff_=0.3 eV, ^b^Ref. [22–25], ^c^Ref. [26–28]

To minimize this systematic error introduced by the influence of *U*_eff_ we decided to keep its value as small as possible, but high enough to yield a band gap in the ground‐state calculation. For ZnFe_2_O_4_ N‐afm, this is the case with *U*_eff_=0.3 eV. A simple shift of the orbital energies applying the SCISSOR correction was abandoned since the Hubbard correction also affects the ground state wavefunction. The dielectric constant calculated for the ground state changes from 17.2 without correction to 14.1 with *U*_eff_=0.3 eV.

**Figure 2:**
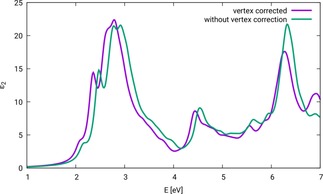
Optical spectra of ZnFe_2_O_4_ configuration N‐afm, calculations carried out with PBE+*U* (*U*_eff_=0.3)/ev*GW*/BSE, with and without vertex correction.

In earlier studies^7^ it has been found that vertex corrections have a significant impact on electronic band gaps and band energies. To test the influence of the vertex correction on zinc ferrite, we performed calculations of the scheme PBE+*U* (*U*_eff_=0.3 eV)/ev*GW*/BSE with and without vertex corrections. The resulting optical spectra can be found in Figure 2. The spectrum including vertex corrections is shifted to lower excitation energies by ∼0.1 eV. The influence of vertex corrections on the intensity increases with increasing excitation energies. This can clearly be seen in the signal near 6.5 eV, where the intensity is notably reduced by the application of vertex correction. Since for the investigation of photocatalytic activity the first excitations are most important, there is overall only a minor effect of vertex correction on the interpretation of results, but it will be included anyway for reasons of physical correctness.

One of the properties of interest of transition metal oxides is the optical band gap. While a *GW* calculation provides the electronic band gap, the optical gap can be obtained by solving the Bethe‐Salpeter equations (BSE). The electronic and optical band gap calculated with the utilized *GW* and BSE approaches can be found in Table 2. As mentioned before, the PBE ground state wavefunction for ZnFe_2_O_4_ has a metallic character. Only self‐consistent *GW* approaches (scGW0 and sc*GW*) are able to open a band gap. Therefore the results for PBE/G0W0 /BSE, PBE/evGW0 /BSE, and PBE/ev*GW*/BSE without Hubbard correction are wrong and thus are excluded from Table 2. Furthermore, in all cases using sc*GW* or scGW0 , the orbital energies of spin‐up and spin‐down channels were different from each other after the vertex correction, which is not reasonable for an antiferromagnetic state. Only the approach PBE/scGW0 /BSE maintained the similarity. Hence, this is the only approach using the self‐consistent *GW* approximation scGW0 which is listed in Table 2, but the result still has to be taken with care. As can be seen from Table 2, the values for the electronic band gap $E_{g}^{elec}$ is dependent on the utilized variant of *GW* as well as on the underlying ground‐state calculation. The electronic band gaps calculated with all approaches using the COHSEX‐approximation are with 2.57 eV for G0W0 , 2.76 eV for evGW0 and 2.84 eV for ev*GW* generally larger than those obtained with the PBE+*U* Ansatz, 2.13 eV for G0W0 , 2.38 eV for evGW0 and 2.48 eV for ev*GW*. This is also true for $E_{g}^{elec}$ calculated with PBE/scGW0 , 2.74 eV. The results for $E_{g}^{elec}$ using evGW0 and ev*GW* are similar to each other, and deviate by 0.08 eV from each other using the COHSEX‐approximation and by 0.1 eV using PBE+*U*. In this case the iteration of the screened Coulomb potential is not critical. G0W0 yields smaller band gaps than evGW0 and ev*GW* in both cases.

The energy of the first excitation with non‐zero oscillator strength and the first maximum in the optical spectrum can be compared to the optical band gap. All methods using PBE+*U* predict the first non‐zero oscillator strength to be the first maximum, which indicates a steep increase in absorption in this energetic region. In all other cases, the difference between first non‐zero oscillator strength and the first maximum ranges from 0.08 to 0.11 eV for the COHSEX approaches and 0.23 eV for PBE/scGW0 /BSE. The predicted excitonic effect, calculated as $E_{g}^{elec}-E_{osz}^{1^{st}-max}$ , is between 0.28 and 0.41 eV and is thus non‐negligible. The excitonic binding energy of transition metal oxides varies between few meV^29^ and several tenth of eV.^30^ Additionally to our results, Table 2 shows experimental results for the optical band gap of ZnFe_2_O_4_ from literature. As can be seen, the results can be divided in two ranges: 1.78–2.01 eV and 2.61–3.25 eV. Because of the inconsistency in experimental results, comparison with experiment does not allow evaluation of the quality of the different methods (cf. Table 2 and Ref. [3]). In an earlier publication,^3^ we predicted the electronic band gap of ZnFe_2_O_4_ with a dielectric dependent self‐consistent hybrid approach to be 2.89 eV. Considering the basis‐set incompleteness error of the earlier approach, all results listed in Table 2, except for PBE+*U*/G0W0, are in agreement with our previous result. Ziaei and Bredow^31^ applied a PBE/G0W0 /BSE approach to ZnFe_2_O_4_, yielding an indirect electronic band gap of 2.02 eV and an optical band gap of 1.93 eV. Different from the present calculations, the authors in Ref. [31] applied norm‐conserving pseudopotentials, that helped probably by error compensation, to obtain a non‐metallic ground‐state with the PBE calculation. Besides this result being in the range of the results in Table 2, the excitonic effect is predicted to be much smaller with 0.09 eV.

**Figure 3:**
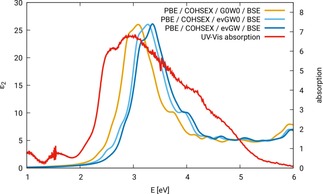
Optical spectra of ZnFe_2_O_4_ configuration N‐afm. BSE calculations carried out based on different variants of *GW* on top of a converged COHSEX calculation, compared to an experimental absorption spectrum, cf. reference [32].

Figure 3 shows the optical spectra of ZnFe_2_O_4_ configuration N‐afm, calculated with G0W0 /BSE, evGW0 /BSE, and ev*GW*/BSE, being based on a converged COHSEX‐*GW* run (cf. lines 1–3 in Table 2). The spectra are compared to the most recent experimental UV‐Vis spectrum of ZnFe_2_O_4_ with a defined degree of inversion of 0.074±0.015. Details on the synthesis and the collection of the spectrum can be found in Ref. [32]. In the literature on spinel ferrites, the degree of inversion is often not indicated. We showed in an earlier publication,^3^ that the degree of inversion critically influences the fundamental band gap of spinel ferrites. Thus, we assume that the optical spectrum is influenced, too. By comparison to an experimental spectrum with a defined and very small degree of inversion, we keep this source of error as small as possible.

As can be seen from Figure 3, the shape of the spectrum does not depend significantly on the underlying *GW* approach. The spectra obtained with the approaches using ev*GW*0 and ev*GW* are almost identical. The most pronounced difference is a slight reduction of intensity for the maximum around 3 eV for ev*GW*, which is due to the iteration of *W*. The spectrum calculated based on the G0W0 approach is slightly shifted to lower excitation energies (∼0.2 eV). All three spectra show a double peak in agreement with experiment, but at significantly higher energies, the difference varying between 0.3 and 0.5 eV.

**Figure 4:**
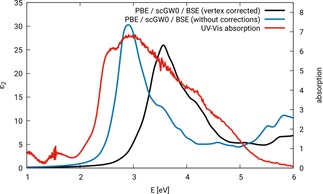
Optical spectra of ZnFe_2_O_4_ configuration N‐afm, calculations carried out with PBE/scGW0 /BSE, with and without vertex corrections, compared to an experimental absorption spectrum, cf. reference [32].

Figure 4 shows the optical spectrum of ZnFe_2_O_4_ configuration N‐afm calculated with the approach PBE/scGW0 /BSE. This method was used by us in an earlier publication,^32^ but at that time vertex correction was not available yet. The vertex correction shifts the spectrum to higher energies, deteriorating the agreement with the experiment. This is most probably due to an elimination of error compensation effects.

**Figure 5:**
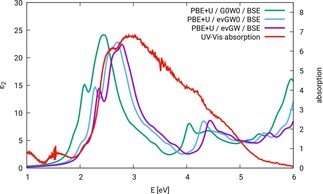
Optical spectra of ZnFe_2_O_4_ configuration N‐afm. BSE calculations carried out on top of different variants of *GW* on a PBE+*U* (*U*_eff_=0.3 eV) ground‐state wavefunction, compared to an experimental absorption spectrum, cf. reference [32].

Figure 5 shows the BSE optical spectra of ZnFe_2_O_4_ configuration N‐afm, on top of G0W0 , evGW0 , and ev*GW*, being based on a PBE+*U* ground‐state wavefunction. As in Figure 3, the spectra yielded by the evGW0 and ev*GW* approach are very similar. The spectrum based on G0W0 is shifted to smaller excitation energies (∼0.2 eV). The double‐peak structure shown by the other spectra around 2.8 eV is not predicted by the G0W0 based spectrum.

By comparing all of the shown spectra in Figures 3, 4 and Figure 5 it is concluded that a best agreement with experimental findings is obtained by using PBE+*U*/evGW/BSE. The shape of the corresponding spectrum resembles the experimental result in particular in the onset region. The onset of both spectra is around 2 eV. The fist sharp maximum of the calculated spectrum around 2.4 eV may be hidden behind the broad signal of the experimental spectrum. The minimum of the double‐peak feature around 2.8 eV is matched by the calculated spectrum. Furthermore, in the region around 4.5 eV the experimental spectrum shows signs of several underlying peaks, which can also be seen in the calculated spectrum. In the comparison to the experimental spectrum it has to be kept in mind, that the degree of inversion of ZnFe_2_O_4_ was with 0.074 very low, but not exactly zero, as in the calculation. As we will see later, the degree of inversion does influence the optical spectrum, so small deviations between experiment and theory are expected. Overall, the position and shape of the spectrum gained by the approach PBE+*U*/ev*GW*/BSE leads us to the conclusion, that this methods is suitable for the treatment of ZnFe_2_O_4_.

The shape of the spectrum shown by Ziaei and Bredow^31^ gained by PBE/G0W0 /BSE resembles the shape of the spectrum gained by PBE+*U*/ev*GW*/BSE presented in this work, but the position of the peaks is different. Assuming that the shape of the spectrum is mainly dependent on the underlying ground‐state calculation, it becomes clear, that the approaches used in Ref.^31^ and in this work are similar. In both cases, a PBE‐based method was used, while in our case the ground‐state wavefunction was forced to represent a semiconductor by introducing a small Hubbard Potential *U*, and in the case of Ref. [31] this was achieved by using norm conserving pseudopotentials. Because of the enhanced physical correctness of ev*GW* including vertex corrections over G0W0 , as well as the good agreement with the experimental spectrum, we are confident that the peak positions of our PBE+*U*/ev*GW*/BSE calculation are reasonable.

**Figure 6:**
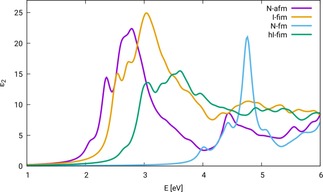
Optical spectra of ZnFe_2_O_4_ configurations N‐afm, I‐fim, N‐fm, and hI‐fim, calculations carried out with PBE+*U*/ev*GW*/BSE, $U_{\mathrm{eff}}^{N-\mathrm{afm}}$ =0.3, $U_{\mathrm{eff}}^{I-\mathrm{fim}}=U_{\mathrm{eff}}^{N-\mathrm{fm}}=U_{\mathrm{eff}}^{\mathrm{hI}-\mathrm{fim}}$ =0.

In 2016, Zviagin et al. published ellipsometric measurements on ZnFe_2_O_4_ thin films,^33^ including a spectral representation of the optical constant. The resulting spectrum, like ours, has its onset around 2 eV, but does not show good agreement, neither to our calculated, nor to the experimental spectrum shown in this work. As stated in Ref. [33], the ZnFe_2_O_4_ films shows ferrimagnetic coupling, probably due to a non‐zero degree of inversion. To investigate the influence of the spin state, we calculated BSE optical spectra of three additional configurations of zinc ferrite. Figure 6 shows optical spectra calculated with PBE+*U*/ev*GW*/BSE for four configurations of ZnFe_2_O_4_:^3^ N‐afm, I‐fim, N‐fm, and hI‐fim. For the three configurations I‐fim, N‐fm, and hI‐fim no Hubbard *U* had to be introduced to open a band gap and the starting point for the ev*GW* calculation is thus plain PBE. As can be seen, cation distribution and spin state influence the shape and position of the optical spectrum. Complete inversion of ZnFe_2_O_4_ leads to a slight overall shift to higher excitation energies, an increased intensity, but the shape of the spectrum is very similar to that of normal, antiferromagnetic (N‐afm) ZnFe_2_O_4_. In the ferromagnetic spin state the first peak is shifted to 4 eV, and the slope of the onset is reduced compared to ZnFe_2_O_4_ N‐afm. The spectrum for the half‐inverse cation distribution with ferrimagnetic coupling (hI‐fim) shows a shift of the onset to 2.7 eV and a reduction of intensity compared to the spectrum of ZnFe_2_O_4_ configuration N‐afm. The configuration hI‐fim resembles the cation distribution and spin configuration of Ref. [33], however the exact degree of inversion of the material in the publication of Zviagin et al. is not clear, while it is exactly 50 % in this work. The corresponding spectra show reasonable agreement. Both spectra show a shoulder below 3 eV. The first maximum of the calculated spectrum is located at 3 eV, while in Ref. [33] it is with ∼3.5 eV located at slightly higher energies. Furthermore, the overall shape of the calculated spectrum resembles the experimental result, having no pronounced minimum between the two groups of maxima around 3.5 eV and 5 eV. Deviations of calculation and experiment are expected, since, as stated above, the degree of inversion of the material in Ref. [33] is not indicated. Additionally, the experiment was carried out with thin films, which can feature surface effects.

The result in Figure 6 shows, that it is important to take into account cation distribution and spin state, when handling ZnFe_2_O_4_. As was shown in Reference [3] antiferromagnetic and ferromagnetic ZnFe_2_O_4_ are very close in energy (2.2 kJ/mol), and the newly examined configuration hI‐fim is – according to our calculations – only 9.3 kJ/mol less stable than configuration N‐afm. According to the results depicted in Figure 6, the wide range of experimental results for the optical band gap of ZnFe_2_O_4_ listed in Table 2 can be explained by the existence of ZnFe_2_O_4_ with different cation distributions and spin states in the measurement samples. The different optical properties have an effect on processes that involve visible light irradiation, like photoelectrochemical water splitting. Due to their large optical gaps, ferromagnetic, as well as half‐inverse ferrimagnetic ZnFe_2_O_4_ will show no (or low) photocatalytic activity.

## 3 Conclusion

In this work, the dependence of the electronic and optical band gap energy and the optical spectrum on the particular variant of the *GW*‐BSE approach as well as the underlying wavefunction were examined for zinc ferrite ZnFe_2_O_4_. As starting points for *GW* we chose plain PBE, PBE+*U*, as well as a combination of PBE and a converged COHSEX approach. For the PBE+*U* approach, the Hubbard potential *U* was chosen to be as small as possible to open a band gap and have a non‐conducting ground‐state wavefunction, to minimize empirism. By comparison to an experimental spectrum of ZnFe_2_O_4_ with a very small degree of inversion, the approach using PBE+*U*/ev*GW*/BSE was shown to be the most suitable for ZnFe_2_O_4_.

The optical spectra of normal antiferromagnetic, inverse ferrimagnetic, normal ferromagnetic, and half‐inverse ferrimagnetic ZnFe_2_O_4_ were compared. The results reveal a pronounced dependency of shape and intensity of optical spectra on the cation distribution and magnetic configuration of ZnFe_2_O_4_. It is expected that these findings can be transferred to other spinel ferrites.

## Supporting information

As a service to our authors and readers, this journal provides supporting information supplied by the authors. Such materials are peer reviewed and may be re‐organized for online delivery, but are not copy‐edited or typeset. Technical support issues arising from supporting information (other than missing files) should be addressed to the authors.

Supplementary
