# Supplementary material for: GW‐BSE Calculations of Electronic Band Gap and Optical Spectrum of ZnFe2O4: Effect of Cation Distribution and Spin Configuration
Source: Chemphyschem. 2020 Feb 12;21(6):546–51. doi: 10.1002/cphc.201901088 (PMC7155046; doi:10.1002/cphc.201901088)
Supplement: Supplementary file 1 — Supplementary [file CPHC-21-546-s001.pdf]

CHEMPHYSCHEM

Supporting Information

GW-BSE Calculations of Electronic Band Gap and Optical Spectrum of $ZnFe_2O_4$: Effect of Cation Distribution and Spin Configuration

Anna C. Ulpe* and Thomas Bredow© 2020 The Authors. Published by Wiley-VCH Verlag GmbH & Co. KGaA.
This is an open access article under the terms of the Creative Commons Attribution License, which permits use, distribution and reproduction in any medium, provided the original work is properly cited.

Table S1: Influence of the plane waves cutoff energy on the optical band gap of $ZnFe_2O_4$

Cutoff energy [eV]	E_g [eV]
450	2.18
600	2.17

Table S2: Influence of the κ-grid on the optical band gap of $ZnFe_2O_4$

κ-grid	E_g [eV]
3×3×3	2.16
4×4×4	2.17

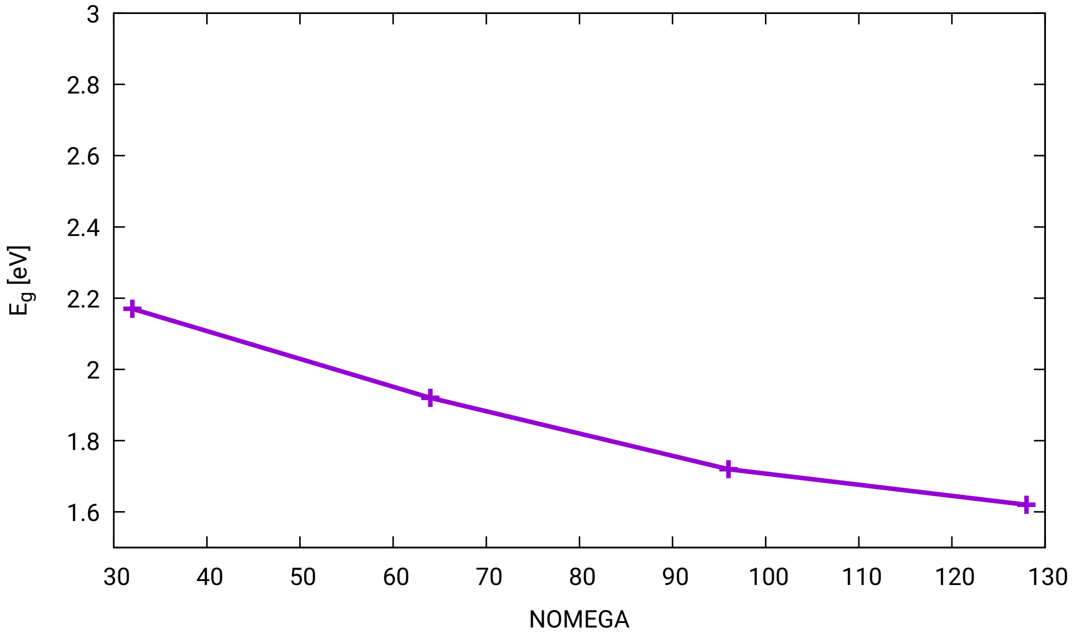

Figure S1: Influence of the number of frequency points (NOMEGA) on the optical band gap of $ZnFe_2O_4$.

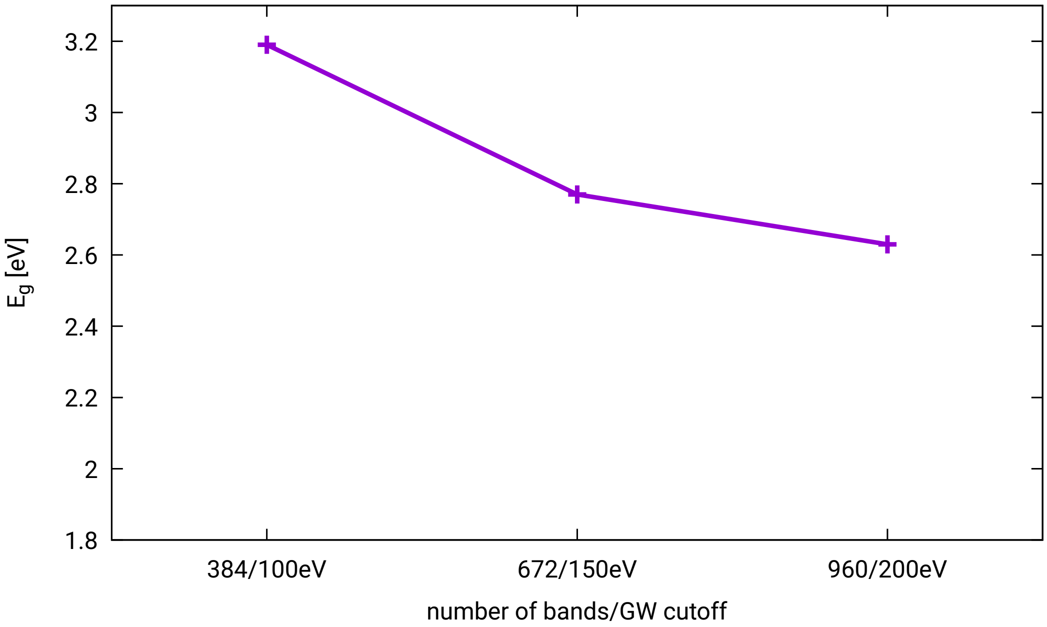

Figure S2: Influence of number of bands and GW cutoff energy on the optical band gap of $ZnFe_2O_4$.

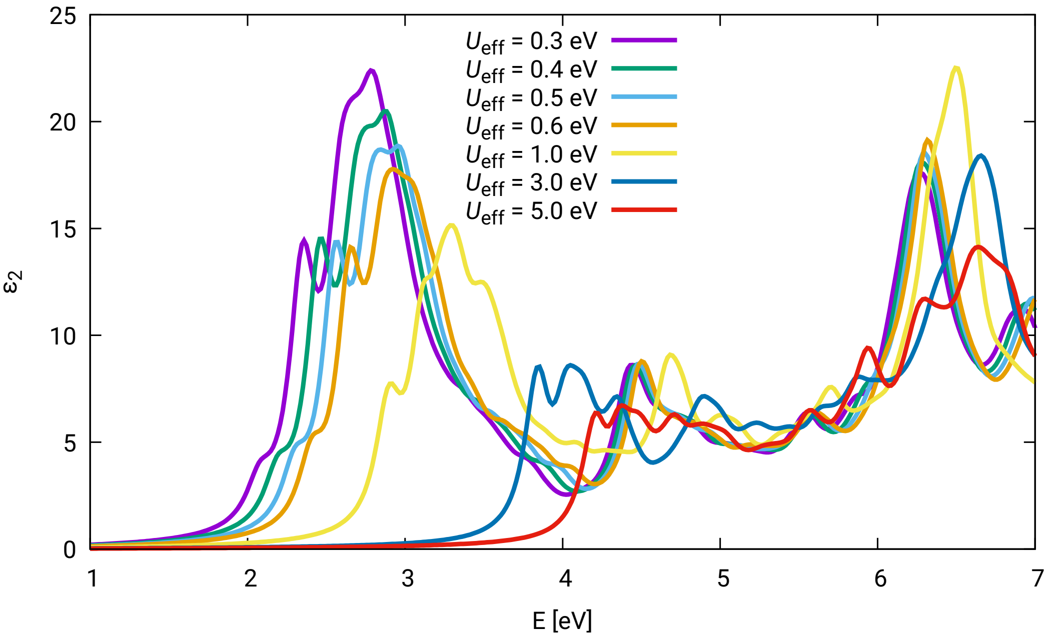

Figure S3: Optical spectrum of $ZnFe_2O_4$ configuration N-afm calculated with PBE+U (U_{eff} = 0.3, 0.4, 0.5, 0.6, 1.0, 3.0, 5.0 eV)/ evGW/ BSE.
